# Editorial: Pediatric infectious diseases and global action plan on AMR

**DOI:** 10.3389/fped.2025.1567171

**Published:** 2025-02-10

**Authors:** Zikria Saleem

**Affiliations:** Department of Pharmacy Practice, Faculty of Pharmacy, Bahauddin Zakariya University, Multan, Pakistan

**Keywords:** children, infectious diseases, global action plan, antimicrobial resistance, peadiatric

**Editorial on the Research Topic**
Pediatric infectious diseases and global action plan on AMR

Antimicrobial resistance (AMR) is a critical global health challenge that disproportionately affects pediatric populations, especially in low- and middle-income countries (LMICs) (1). Despite the Global Action Plan on AMR put forth by the World Health Organization (WHO), which emphasizes five strategic objectives—raising awareness, strengthening surveillance, infection prevention, optimizing antimicrobial use, and sustainable investment—pediatric considerations remain underrepresented (1, 2). Healthcare-associated infections (HAIs) impose a significant burden in LMICs, where limited resources, inadequate infection prevention measures, and overcrowded healthcare facilities contribute to higher rates of morbidity and mortality among children (3). Neonatal sepsis caused by multidrug-resistant pathogens is a major concern, with resistance rates reaching as high as 90% in intensive care units in some regions (4). In LMICs, where antibiotic use in pediatric care often approaches more than 80%, the lack of targeted interventions contributes to escalating resistance rates (5). This Research Topic, “Pediatric infectious diseases and global action plan on AMR,” brings together seven studies that highlight the pressing need to integrate pediatric-specific approaches into AMR strategies, while addressing the unique challenges in LMICs.

Sheikh et al. conducted a point prevalence survey across 23 pediatric wards in Punjab, Pakistan, revealing an antibiotic use prevalence of 82.1%, with most prescriptions falling under the WHO Action, Watch, Reserve (AWaRe) classification's Watch category. Only 2% of prescriptions were culture-based, underscoring the need for robust stewardship programs to optimize antibiotic use. Wang et al. provided a 20-year epidemiological analysis of pediatric tuberculosis in Southwest China, highlighting disparities in TB prevalence among ethnic minorities and the need for public health investments to address these inequities. Li et al. analyzed respiratory tract infections in Southern Sichuan, China, identifying age- and gender-specific pathogen trends and emphasizing co-infections as significant risk factors for pneumonia. In Ethiopia, Yilma et al. assessed infection prevention and control (IPC) practices among healthcare workers in a pediatric department, finding that only 50.4% demonstrated good IPC practices. Similarly, Adbela et al. examined pneumonia outcomes among pediatric patients in Eastern Ethiopia, identifying malnutrition, unvaccinated status, and complicated pneumonia as key risk factors for mortality. Alnezary et al. investigated the management of pediatric diarrhea by community pharmacists in Saudi Arabia, revealing inadequate practices and a high prevalence of inappropriate antimicrobial dispensing. Their study highlights the need for pharmacist training programs to improve counseling and reduce AMR. Finally, Yehuala et al. leveraged machine learning algorithms to predict acute respiratory infections (ARI) and identify determinants in Sub-Saharan Africa.

Together, these studies underscore the urgency of integrating pediatric-specific strategies into the WHO Global Action Plan's framework. Raising awareness through targeted education, strengthening surveillance systems with data-driven tools, enhancing preventative measures, and optimizing antimicrobial use are steps that are critical to combating AMR in children, such as early de-escalation based on culture results and adherence to WHO guidelines. Sustainable investment in public health infrastructure, improved diagnostics, increased pediatric-specific antibiotic research, better formulations, and rational pharmaceutical policies, particularly in LMICs, are essential to ensure equitable access to interventions. This collection of research highlights the pivotal role of multidisciplinary efforts by engaging stakeholders from child health in addressing AMR and safeguarding the health of vulnerable pediatric populations worldwide. The insights presented herein provide a robust foundation for future research and policy initiatives to combat AMR in children effectively.

## References

[1] Romandini A Pani A Schenardi PA Pattarino GAC De Giacomo C Scaglione F. Antibiotic resistance in pediatric infections: global emerging threats, predicting the near future. Antibiotics. (2021) 10:393. 10.3390/antibiotics10040393 33917430 PMC8067449

[2] Pana ZD El-Shabrawi M Sultan MA Murray T Alam A Yewale V Fighting the hidden pandemic of antimicrobial resistance in paediatrics: recommendations from the international pediatric association. BMJ Paediatrics Open. (2023) 7(1):e002084. 10.1136/bmjpo-2023-002084 37500294 PMC10387713

[3] Arif S Sadeeqa S Saleem Z Latif S Sharif M. The burden of healthcare-associated infections among pediatrics: a repeated point prevalence survey from Pakistan. Hosp Pract. (2021) 49:34–40. 10.1080/21548331.2020.1826783 32990488

[4] Okomo U Akpalu EN Le Doare K Roca A Cousens S Jarde A Aetiology of invasive bacterial infection and antimicrobial resistance in neonates in sub-saharan Africa: a systematic review and meta-analysis in line with the STROBE-NI reporting guidelines. Lancet Infect Dis. (2019) 19:1219–34. 10.1016/S1473-3099(19)30414-1 31522858

[5] Hafeez M Saleem Z Bukhari NA Hussain K Shamim R Hussain A Off-label antibiotic use in a specialized children care hospital in Punjab, Pakistan: findings and implications. J Infect Dev Ctries. (2020) 14:540–4. 10.3855/jidc.12058 32525842

